# Supplementary material for: Host immune players and their response to Hepatitis C therapies
Source: PLOS Glob Public Health. 2024 Jun 12;4(6):e0003110. doi: 10.1371/journal.pgph.0003110 (PMC11168669; doi:10.1371/journal.pgph.0003110)
Supplement: Attachment — Submitted filename: Response to Reviewers.docx [file pgph.0003110.s002.docx]

**Cover letter**

We are very grateful for the insightful comments provided by the editor and each of the external reviewers of this manuscript. The thorough review helped immensely in the shaping of the manuscript. The suggestions and comments have been closely followed and revisions have been made accordingly. The suggested changes to the manuscript are indicated in the text by using track changes.

**Response to the Reviewer’s Comments**

**Reviewer 1:**

**Major corrections:**

1. Lines 13-15. The sentences "IL-11 expression... inhibiting the Th1 response" and "The decrease in IL-11.... restoration of Th1 response..." seem to suggest, on the one hand, that low IL-11 is associated with inhibition of Th1 response and, on the other hand, that low IL-11 is important for the Th1 response restoration. Which one is it?

**Response:**

Thank you for your suggestion. The response has been addressed and changes have been incorporated under the abtract heading on lines 12-14.

2. Lines 71-73. There needs to be a clear justification for why the authors look at those five genes and not others. Not wanting to limit the genes queried to inflammatory markers is fine. Still, the reason why the authors selected those specific genes should be presented in the introduction or result section.

**Response:**

We acknowledge that inlcusion of these lines was important to avoid any ambiguity and concern as raised by the reviewer as well; few lines justifying the use of these genes in the study have been included under the introduction section on page 2-3 lines 64-69.

3. Lines 141-143: If you are not assuming a Gaussian distribution, you should use a non-parametric test like a Wilcoxon rank-sum test over a Student t-test (a t-test is a parametric test and assumes that the data is normally distributed).

**Response:**

Thank you for your suggestion. A statistician was consulted for the statistical analysis. For the data of any group the outliers were removed and all data has already been normalized against housekeeping gene making it normally distributed therefore student t test as applied, can be used for statistical analyses.

4. Line 143: There is no correction for multiple testing anywhere in the paper.

**Response:**

Thank you for your comment. Kindly clarify statement we could not understand the point of esteemed reviewer.

5. Line 144: There is no description of the clinical characteristics of the cohort (e.g., sex, age of the participants) other than the number of participants by treatment group (presented in Figure 1). A table with those clinical parameters stratified by treatment groups should be given as Table 1.

**Response:**

We acknowledge that inlcusion of this demographic table was important to avoid any ambiguity and concern as raised by the reviewer as well. So although it was not included initially, now as per suggestions, it is included in supplementary data as table 3.

**Table 1. Demographic and basic clinical attributes of the enlisted patients** (* P values < 0.05 were considered significant)

| **Variable** | **Total**  **(n = 368)** | **Responders**  **(n = 241)** | **Non-responders**  **(n = 127)** | **P value*** |
| --- | --- | --- | --- | --- |
| **Gender (Male/Female)** |  | 98/143 | 47/80 | 0.496 |
| **Baseline viral load range (IU/ml)** |  | 2x10^3^-1x10^7^ | 1x10^3^-2x10^7^ |  |
| **Age, year (average ± SD)** |  | 39 ± 15 | 38 ± 16 | 0.459 |
| **ALT, IU/L (average ± SD)** |  | 76± 77 | 103 ± 89 | **<0.001** |

6. Lines 145-146: "Investigations explores...and immune system functions." what does this sentence mean?

**Response:**

We acknowledge the response from the reviewer. The said changes have been incorporated in the manuscript on page 6 lines 151-152.

7. Lines 147, 268, 271: looking at the RNA expression of cytokines does *not* mean looking at the "immune regulatory activity" of those cytokines; do not overinterpret your work.

**Response:**

Thank you very much for your suggestion. The Suggestive changes have been made wherever applicable in the manuscript on page 6 lines 151-152, page 7 lines 156-157 and page 14 lines 329-330.

8. Line 148: Indicate when the samples were collected after initiation of the treatments.

**Response:**

We acknowledge that inlcusion of this information was important to avoid any ambiguity and concern as raised by the reviewer as well. These patients had received the standard IFN/ribavirin treatment (SIT) or pegylated IFN/ribavirin (PEG) or Sofosbuvir (SOFO) (Sovaldi®, Gilead Sciences, Inc) treatments afterwards the samples were collected. The samples were collected at variable time points post treatments as per their visits the hospital the average time varied between 1 to 6 months post treatment.

9. Lines 157, 176: It is not "insignificant"; it is "not statistically significant".

**Response:**

We appreciate your response; the said changes have been incorporated in the manuscript.

10. Line 159: The sentence "In case of PEG, FAM26F expression was upregulated in RVR, ETR" is not supported by stats, so you should indicate that this is only a trend.

**Response:**

The correction is acknowledged. The specific lines have been incorporated in the manuscript.

11. Line 163: Cite only Fig2C, there is no SOFO in Fig 2B.

**Response:**

The correction is acknowledged. We have corrected this mistake in the manuscript.

12. Line 166-171: Mention that nothing reaches statistical significance (or add the stats to Figure 3).

**Response:**

The esteemed reviewer is correct in pointing this issue. Statistics was applied on all figures among all groups however asterisk (*) and p values are shown only for significant differences. Since figure 3 was statistically not significant so no asterisk (*) were mentioned now same is mentioned in figure legends. The groups were compared however no statistically significant difference was found. You can find more details, including p-values, in Table 2 of the additional data. The said changes have been incorporated in the manuscript on page 8 lines 176-177.

13. Line 184: "significant downregulation of RVR compared to ETR". That does not match the stats shown in Figure 4C.

**Response:**

I appreciate your feedback. Although this point has been previously highlighted in the manuscript, it's noteworthy that the expression levels of SOFO were significantly downregulated at RVR compared to ETR, as illustrated in Figure 4C. Additional details, including the p-value information, can be found in Table 2 of the supplementary data with serial number 48, and we have reiterated its value below for reference.

| 48 | RVR SOFO IL7 vs ETR SOFO IL7 | ** | Yes | 0.0074 |
| --- | --- | --- | --- | --- |

14. Line 187-191: There is no stat indicated in Fig 5.

**Response:**

I appreciate your comment. It's important to note that while the comparison between groups did not yield statistical significance, the absence of significance indicators is due to the non-mention of signs, as error bars themselves indicate the application of statistical analysis. Further information, including the p-value details, can be referenced in Table 2 of the supplementary data. However we now mentioned in text nothing is found statistical significant.

15. line 196: "downregulated significantly for ETR" that doesn't match the stats in Figure 6A.

**Response:**

The esteemed reviewer is correct in pointing this issue.The said changes have been incorporated in the manuscript.

16. Lines 206-207: "RVR...significantly lower than control/TN". There is no statistical support for RVR vs control/TN, according to Fig 6C.

**Response:**

The correction is acknowledged. We have corrected this mistake in the manuscript.

17. Line 222: Do you mean "IL15"? IL11 is written in the text.

**Response:**

We appreciate your response; we have corrected where it was mentioned in the manuscript.

18. Line 230: The stats in the text do not match the stats in Fig. 8B and Fig. 8C.

**Response:**

The correction is acknowledged. We have corrected this mistake in the manuscript.

19. Line 238: No stat is shown in Fig. 9.

**Response:**

Thank you for your comment. Statistics was applied on all figures among all groups however asterisk (*) and p values are shown only for significant differences. Since figure 9 was statistically not significant so no asterisk (*) were mentioned now same is mentioned in figure legends. The groups were compared however no statistically significant difference was found. You can find more details, including p-values, in Table 2 of the additional data.

20. Line 246: There is no statistical support for IL27 being lower in ETR compared to TN in Fig 10B.

**Response:**

Thank you for your comment. Statistics was applied on all figures among all groups however asterisk (*) and p values are shown only for significant differences. Since figure 10B was statistically not significant so no asterisk (*) were mentioned now same is mentioned in figure legends. I want to highlight that when we examined the groups, we didn't identify any notable statistical differences.

21. Line 388: Add a paragraph on the limitations of the study.

**Response:**

We appreciate your response, the said change have been incorporated on page 20 lines 452-455.

22. All figures show individual points for each participant, not just average expressions and error bars. Replace bar plots/histograms with jitter plots. Add axis labels. Indicate in the legend what the *, **, *** correspond to for the p-value you calculated. Put the legends of the figures at the end of the document and not in the text.

**Response:**

We acknowledge reviewer’s comment. The use of jitter plots and bar plots doesn't affect the statistical interpretation of the results; it's simply an alternative way of presenting the data. Therefore, we have chosen to represent it using bar plots.

**Minor corrections:**

Line 7: add between these "treatment" groups.

**Response:**

The word “these” has been replaced with word “treatment” in abstract line7.

Line 9: indicates that those genes were looked in blood in the abstract.

**Response:**

We acknowledge reviewer’s comment, and the missing information has been added in abstract on lines 9.

Line 106: replace "digit" by "number".

**Response:**

The word number has been introduced on page 5, line 106 as per suggestions of the reviewer.

Lines 108-109: replace "the treatment points when blood sample were drawn" by "the number of samples collected for each treatment response group".

**Response:**

The statement has been introduced as per suggestions of the reviewer.

Lines 119, 131: Use qRT-PCR over QRT-PCR. Be consistent with the choice of capitalization across the article (ex. line 132 you use qRT-PCR).

**Response:**

The correction is acknowledged. The whole manuscript was critically scrutinized and QRT-PCR is now consistently used in the document.

Fig 2a: The horizontal bar for the comparison TN vs "SVR R" should go all the way to the halfway point of the SVR R bar.

**Response:**

Thanks for the comment. The said changes have been incorporated.

Line 181: add that you are comparing RVR vs TN. Was the upregulation of IL7 significant?

**Response:**

Thank you for your opinion. Change in expression in RVR from TN was not found statistically significant. We have included the suggested changes on page 8 lines 199-200.

Lines 225, 249: replace "than" by "compared to".

**Response:**

Instead of than it has been written compared to on page 10 lines 225, 249.

Line 259: Table 2 is useful, but it would be easier for the reader if the stats are indicated in the figures.

**Response:**

Thank you for your comment. The figures contain self-explanatory statistics, and for additional verification, you can refer to Table 2 in the supplementary data.

**Reviewer #2:**

1. Collect the figure ligands in a separate part after the references

**Response:**

Thank you for your suggestion. Figure legends have now been properly separated from the text and added after the references.

2. At RNA extraction and purity method: A pretreatment of the extracted RNA with DNAase should be done in order to eliminate any DNA contamination during the extraction process especially when using Trizol for RNA extraction. Otherwise the primer design should be done so primers have to span an exon – exon junction. Did you check this point?

**Response:**

We appreciate your response; we have used DNAase treatment before quantification. 10 µl of 10X DNAse buffer was used to prevent DNA contamination. The said response has been incorporated on page 5 lines 125-126.

3. The primers for FAM26F do not have any match at primer blast.

**Response:**

Thank you for your response. Each primer set was checked for specificity using Primer-BLAST tool on the NCBI server (1). Primer-BLAST was used in its default setting where the primers were checked within the ‘nr’ database.

1- Ye, J. et al., 2012. Primer-BLAST: a tool to design target-specific primers for polymerase chain reaction. BMC bioinformatics, 13, p.134. Available at: http://www.ncbi.nlm.nih.gov/pubmed/22708584 [Accessed September 12, 2016].

4. What is the role of the IDO-1 primers in this study

**Response:**

This primer pair has been removed from the list.

5. You should mention the method of quantification of your genes and in your case it should quantified by relative fold change obtained by the 2−ΔΔCt method.

**Response:**

Thank you for your comment.There are different methods of quantification and copy number is also one of valuable method. mRNA levels were calculated as copy number relative to 100 copies of GAPDH. The relativistic expression (rE) of target gene was determined as:

rE = 100 x 2-ΔCt

Where, ΔCt = mean Ct (target gene) minus mean Ct (GAPDH)

This method is reported in various research reports (1-4).

1) Mussil B, Sauermann U, Motzkus D, Stahl-Hennig C, Sopper S. Increased APOBEC3G and APOBEC3F expression is associated with low viral load and prolonged survival in simian immunodeficiency virus infected rhesus monkeys. Retrovirology. 2011 Sep 28;8:77. doi: 10.1186/1742-4690-8-77. PMID: 21955401; PMCID: PMC3192745.
2) Javed A, Leuchte N, Neumann B, Sopper S, Sauermann U. Noncytolytic CD8+ Cell Mediated Antiviral Response Represents a Strong Element in the Immune Response of Simian Immunodeficiency Virus-Infected Long-Term Non-Progressing Rhesus Macaques. PLoS One. 2015 Nov 9;10(11):e0142086. doi: 10.1371/journal.pone.0142086. PMID: 26551355; PMCID: PMC4638345.
3) Jabeen, K. Malik, U. Mansoor, S. Shahzad, Sh.Zahid, S. Javed, A. 2021 “Effect of Oxidative Stress and Calcium Deregulation on FAM26F (CALHM6) Expression during Hepatitis B Virus Infection.” BMC Infectious Diseases, [https://bmcinfectdis.biomedcentral.com/articles/10.1186/s12879-021-05888-0 – 2021](https://bmcinfectdis.biomedcentral.com/articles/10.1186/s12879-021-05888-0%20–%202021)
4) Jabeen K, Javed A, Waris A, Shahzad Sh. Restoration of IL-11 and IL-15 cytokine production post calcium modulators and ROS treatment can assist viral clearance both in vitro and in vivo. Iran J Basic Med Sci 2023; 26: 1-7. <https://pubmed.ncbi.nlm.nih.gov/36742132/>

6. The figures for gene expression should be measured in fold of expression not in copy number as in the link according the 2−ΔΔCt equation: https://bmcgenomics.biomedcentral.com/articles/10.1186/1471-2164-11-455/figures/3

Then repeat the statistical analysis for your study.

**Response:**

Thank you for your suggestion. mRNA levels can be assessed either in terms of fold expression or copy number. Statistically, both approaches are equally dependable, and the results remain consistent regardless of whether we calculate values in fold change or copy number. mRNA levels were calculated as copy number relative to 100 copies of GAPDH. The relativistic expression (rE) of target gene was determined as:

rE = 100 x 2-ΔCt

Where, ΔCt = mean Ct (target gene) minus mean Ct (GAPDH)

This method is reported in various research reports (1-4).

1) Mussil B, Sauermann U, Motzkus D, Stahl-Hennig C, Sopper S. Increased APOBEC3G and APOBEC3F expression is associated with low viral load and prolonged survival in simian immunodeficiency virus infected rhesus monkeys. Retrovirology. 2011 Sep 28;8:77. doi: 10.1186/1742-4690-8-77. PMID: 21955401; PMCID: PMC3192745.
2) Javed A, Leuchte N, Neumann B, Sopper S, Sauermann U. Noncytolytic CD8+ Cell Mediated Antiviral Response Represents a Strong Element in the Immune Response of Simian Immunodeficiency Virus-Infected Long-Term Non-Progressing Rhesus Macaques. PLoS One. 2015 Nov 9;10(11):e0142086. doi: 10.1371/journal.pone.0142086. PMID: 26551355; PMCID: PMC4638345.
3) Jabeen, K. Malik, U. Mansoor, S. Shahzad, Sh.Zahid, S. Javed, A. 2021 “Effect of Oxidative Stress and Calcium Deregulation on FAM26F (CALHM6) Expression during Hepatitis B Virus Infection.” BMC Infectious Diseases, [https://bmcinfectdis.biomedcentral.com/articles/10.1186/s12879-021-05888-0 – 2021](https://bmcinfectdis.biomedcentral.com/articles/10.1186/s12879-021-05888-0%20–%202021)
4) Jabeen K, Javed A, Waris A, Shahzad Sh. Restoration of IL-11 and IL-15 cytokine production post calcium modulators and ROS treatment can assist viral clearance both in vitro and in vivo. Iran J Basic Med Sci 2023; 26: 1-7. <https://pubmed.ncbi.nlm.nih.gov/36742132/>
